# Effects of CBD (Cannabidiol) on the physiology of Nile tilapia (*Oreochromisn niloticus*) as a chronic stress mitigating agent *In-vivo*

**DOI:** 10.1371/journal.pone.0290835

**Published:** 2023-09-15

**Authors:** Asif Mortuza, Nahian Fahim, Malaika Ahmed, Ahmed Mustafa

**Affiliations:** Department of Biology, Purdue University Fort Wayne, Fort Wayne, Indiana, United States of America; Sher-e-Kashmir University of Agricultural Sciences and Technology of Kashmir, INDIA

## Abstract

This study evaluates the effects of Cannabidiol (CBD) on the physiology of stressed and non-stressed Nile tilapia, reared in a recirculating aquaculture system. Tilapia were fed with and without CBD (0.001% of feed weight) and with and without hydrocortisone stress hormone (0.01% of body weight) every day for four weeks. This experiment compared the plasma cortisol, blood glucose and protein levels, liver and spleen somatic indices (HSI and SSI, respectively), and lysozyme activity of the fish. Stress group (S) had a significantly higher value than the control group (C) in two of the parameters, glucose and lysozyme activity, this is an indication of stress. CBD had a stress reducing effect under stressed conditions in lysozyme activity. Although not significant, the stress reducing effect of CBD on stress biomarkers such as glucose and HSI also seemed promising. Further investigation into the matter may not just be useful in stress mediation in aquatic organisms but may also have implications in human medicine as well.

## Introduction

Due to extensive husbandry methods used in aquaculture, stress in fish is a significant issue. Crowding, handling, and vaccinating are common husbandry methods that can stress the fish, leading to lower productivity [1]. To mitigate the stress response, farmers often use antibiotics and chemical drugs. When antibiotics and other chemical drugs are released into the environment it can lead to the creation of superbugs or it may affect non-target species. The exposure of antibiotics and other chemicals also have the potential to affect human health if the chemicals remain within the fish when consumed. To diminish the use of harmful substances in aquaculture, we must understand the root of the problem, “stress”. Stress, unfortunately, is very common in every form of animal farming, especially aquaculture. Poor stress management causes a loss in productivity due to disease and poor health, this in turn causes farmers to lose thousands of dollars every year. Stress is known as a state of decreased fitness or as a response to any external stimulus that disrupts homeostasis and therefore threatens survival [2]. Chronic stress can make an organism susceptible to various secondary diseases since prolonged exposure to stress has been shown to weaken immune and inflammatory responses [3].

According to Moyle et al. (1990), widespread physiological changes occur within an organism when it is exposed to stress. These responses are known as the primary, secondary, and tertiary stress responses. The primary response is triggered after a stressor is detected by the organism, causing the central nervous system to signal for the release of cortisol and adrenaline into the blood [4]. In the secondary stress response, hematological changes start to occur such as, elevated blood glucose (hyperglycemia) and reduced blood clotting time [4]. Diuresis and electrolyte loss are also a part of the secondary response, causing osmoregulatory dysfunction. The tertiary stress response is characterized by major physiological changes within the body such as, reduced resistance to infectious diseases, reduced growth, reduced reproductive success, and the reduction of survival. We can measure the changes that occur within each stage to determine the level of stress an organism has endured.

To help diminish the use of these harmful substances to lessen the effects of stress in aquaculture, scientists are on the search for alternative solutions such as nutraceuticals. Nutraceuticals are known as functional foods (often phytochemicals) that have been shown to have medicinal properties [5]. Nutraceuticals have repeatedly been shown to potentially alleviate stress and boost the immunity in many different animals including fish [6–8]. Cannabidiol (CBD) is a fairly new nutraceutical that may have the potential to mitigate the stress response. CBD, like tetrahydrocannabinol (THC) is a derivative of the marijuana plant, *Cannabis sativa*. However, unlike THC, CBD is a non-psychoactive compound of the plant. CBD has been shown to be a neuroprotectant [9], alleviate anxiety, psychosis [10] epilepsy [11], depression [12], and pain [13]. Research has also shown that CBD may also benefit the immune system by reducing inflammation [14], cancer [15, 16] and bacterial infections [17]. In 2018, Viudez-Martínez et al. studied the effect of CBD on different gene targets of the hypothalamus-pituitary-adrenal (HPI) axis of mice under control and stress conditions. They did this by administering CBD intraperitoneally in dosages of 5 mg/ kg, 15 mg/kg, or 30 mg/kg (of mice body weight), before the mice were exposed to restraint stress. Using real time PCR analysis, they were able to measure the relative gene expression of corticotropin-releasing factor. Their research showed that when CBD is given in the 5 mg/kg and 15 mg/kg doses, it was able to block the gene expression caused by acute stress. Expression of stress related genes are certain to have a physiological impact on the whole organism’s stress response [18].

Research has been conducted on the widespread therapeutic usage of CBD but there is a lack of research looking at the effect of CBD on the stress physiology of vertebrates. Would CBD have the ability to mitigate stress responses? Would it be able to compensate for the reduction in growth that is seen in fish exposed to high stress conditions in aquaculture? In this experiment, we chose Nile tilapia, *Oreochromis niloticus* as one of the most common fish models used in aquaculture [19, 20]. Overall physiological and immunological responses of fish are similar to that of other vertebrate animals. Their physiological and immunological responses are controlled by the nervous and endocrine system, which are also comparable to other vertebrate animals, making them a good model to study human medical applications.

In our experiment, we tested the effect of CBD on mitigating the chronic stress response of Nile tilapia. To determine its effect, we measured plasma cortisol (primary stress biomarker), blood glucose levels plasma protein levels, liver and spleen somatic indices (secondary stress biomarker), and lysozyme activity (tertiary stress biomarker) in both stressed and non-stressed O. *niloticus*.

## Materials and methods

### Fish maintenance

Nile tilapia, *Oreochromis niloticus*, fingerlings (average length 20±2 cm; average weight 175±10 g) were purchased from Troyer Aqua Farms, Geneva Indiana. The fish were kept in a recirculating aquaculture system (Aquatic habitats ™, Aquatic Eco-Systems, INC). The system was maintained and cared for following an approved animal care protocol (pH: 6.0–7.0, ammonia: <0.05 mg/ L; dissolved oxygen 5.00–7.00 mg/ L, and temperature: 25 ± 2°C). The fish were fed 1.5% of their body weight twice a day with a compatible commercial fish feed, Purina^®^ AquaMax^®^ Fingerling Starter 300 (Purina Mills, MO, USA).

### Ethics statement

All fish were taken care of following an approved protocol by Purdue University Animal Care and Usage Committee (PACUC) following the guidelines of the US National Research Council’s "Guide for the Care and Use of Laboratory Animals".

### Experimental design

No previous studies were found showing the potential effects of CBD on chronic stress physiology of *O*. *niloticus*. Due to this void, the therapeutic dosage for CBD for fish is unknown. To determine the appropriate concentration of CBD, we conducted a preliminary acute study for 72 hours. In the acute study, the fish were maintained following similar experimental design and protocol as this study. However, it was done only over a period of 72 hours and the fish were fed with feed supplemented with three different concentrations of CBD, with and without hydrocortisone. At the end of the experiment we collected blood from the fish and evaluated stress biomarkers such as blood glucose, hematocrit, and plasma protein. From the study we found that there was no significant difference in the stress physiology of *O*. *niloticus* between 0.001%, 0.002%, and 0.003% CBD (% of feed weight) over 3 days. Therefore, in our chronic study, the lowest concentration was tested to see its effect on chronic stress physiology as it is the most economic option for aquaculturists.

For the chronic study, the fish were divided into four different groups. Each group consisted of 4 fish, each fish in individual tanks (individual replicates). Each group was fed with a different diet and were fed twice a day. Group 1 was fed a diet the standard commercial feed (C = Control feed); group 2 was fed with 0.001% (feed weight) CBD (99% pure isolate from Sigma Aldrich^®^, MO, USA) supplemented commercial feed (CCBD = Control feed with CBD); group 3 was fed with 98% hydrocortisone (at 0.01% body weight from Acros Organics, NJ, USA) supplemented commercial feed (S = Stress feed); and group 4 was fed with 0.001% CBD and hydrocortisone (0.01% body weight) supplemented commercial feed (SCBD = Stress feed with CBD). Once hydrocortisone is ingested, the body metabolizes it into cortisol, which is responsible for the stress response. The amount of hydrocortisone used was determined using the findings of previously done research at this concentration [21]. The addition of hydrocortisone in the feed gave us the ability to compare the effects of CBD on stressed and non-stressed *O*. *niloticus*.

### Feed preparation

Since the amount of feed needed depended on the weight of the fish, the feed was prepared weekly. The feed was prepared by dividing the total amount of commercial feed (Purina^®^ AquaMax^®^ Fingerling Starter 300) needed that week in half. One half of the feed would get the calculated dosage of hydrocortisone (0.01% of fish body weight) and thoroughly mixed. The feed was then left out overnight to dry. Then the CBD supplemented fish feed was prepared by further dividing the feed (with and without hydrocortisone) in half. One half of the feed without hydrocortisone and one half of the feed containing hydrocortisone would get 0.001% (of the feed weight) of 99% pure isolate Cannabidiol from Sigma Aldrich^®^. All the feed was allowed to dry overnight and then stored at 4°C. Drying overnight was needed since ethanol was used throughout as a solvent to dissolve and thoroughly mix the CBD and hydrocortisone to the feed. The drying in an unsealed container allowed the ethanol to evaporate out of the feed. A Proximate composition of feed ingredients is presented in Table 1.

**Table 1:** Proximate composition of feed ingredients used to prepare four treatment groups of fish feed represented by C (Control feed), CCBD (Control feed with CBD), S (Stress feed = feed with hydrocortisone) and SCBD (Stress feed with CBD = feed with hydrocortisone and CBD) groups.

Ingredients (% Feed Weight)	C	CCBD	S	SCBD
Crude protein	50	50	50	50
Crude fat	16	16	16	16
Crude fiber	3	3	3	3
Calcium (Ca)	5.2	5.2	5.2	5.2
Phosphorus (P)	1.3	1.3	1.3	1.3
Sodium (Na)	0.6	0.6	0.6	0.6
CBD	‐‐	0.001	‐‐	0.001
Hydrocortisone*	‐‐	‐‐	0.01	0.01

*% Body Weight

### Fish sampling

To calculate the stress biomarkers, the fish were sampled at the end of a four-week period. The fish were euthanized by mixing tricaine methane sulfonate (MS-222) (Western Chemicals, WA) with water at 400 mg/ L. This concentration allows immediate immobilization of the fish to reduce any stress from handling (all within two minutes of catching the fish). The fish were weighed, length was measured (results not included in this paper) and blood samples were collected from the caudal vein using heparinized syringes to prevent blood clot. The blood was placed in a 1.5 mL Eppendorf tube and immediately placed on ice until needed.

The fish were then dissected using aseptic techniques to remove the liver and the spleen. Upon removal, the liver and the spleen were weighed. Then the following parameters were measured using the collected blood and the tissue samples.

### Plasma cortisol

Cortisol is one of the major stress hormones released by the interrenal cells of the head kidney tissue of the fish and thus indicative of stress [21]. The blood was centrifuged at 5000 rpm for 10 minutes to collect the plasma. The supernatant plasma was collected and preserved at -80°C for future use. Due to the small amount of serum that could be isolated, the serum from all the fish within a treatment group (n = 1) was pooled together. To measure the cortisol level in the plasma, Cayman Chemical Cortisol ELISA kit (Item No. 500360) (Ann Arbor, MI, USA) was used, following manufacturer’s protocol.

### Blood glucose

Blood glucose is another measure of stress as changes in blood glucose occur due to increased respiration, decreased metabolic activity and decreased immunity, all indicative of stress [22]. A Glucometer (Freestyle, Abbott Diabetes Care, Inc., Alameda, CA, USA) was used to measure the blood glucose according to the manufacturer’s protocol [23].

### Total plasma protein

Total plasma protein is a good indicator of health and stress levels [24]. As part of the stress response, increased synthesis of certain proteins is induced to act as molecular chaperones to reconstruct damaged cells [25]. A protein refractometer was used to measure the refractive index of all the dissolved solids in the solution. The refractive index scale was 1.333 to 1.360 and the refractometer was calibrated using PBS. To take a reading, a few drops of plasma was placed on the prism and read from the far-right scale in g/ 0.1 L.

### Hepatosomatic index (HSI)

Energy in fish is stored in the muscles as well as the liver in the form of glycogen. During growth and high stress conditions, glycogen is mobilized in the presence of glucagon released by the pancreas. The glycogen storage of the liver is broken down to glucose and released in the blood, affecting the overall size and mass of the liver. Thus, hepatosomatic index (HSI) is a good indicator of nutrition, growth and stress levels of fish [26]. Hepatosomatic index compares the weight of the liver of the fish in proportion to its body weight. The collected liver from the fish were weighed and the following formula was used to measure the HSI of the fish.


HSI=(LiverWeight(g)/BodyWeight(g))×100

### Spleen somatic index (SSI)

Lymphocytes and blood cells are stored in the spleen and mobilized to immunomodulate [27]. Therefore, the spleen somatic index is a good indicator of overall health of fish. Spleen somatic index compares the weight of the spleen of the fish in proportion to its body weight. The collected spleen from the fish were weighed and the following formula was used to find the SSI of fish:

SSI=(SpleenWeight(g)/BodyWeight(g))×100

### Lysozyme activity assay (LAA)

Lysozyme is an enzyme with the ability to lyse bacterial cells, leading to a change in the opacity of a given bacterial solution. This assay aims to assess the endogenous lysozyme’s capability in the fish’s blood to clear the color of the bacterial solution, using a spectrophotometer [28]. As more bacteria are killed, the solution becomes clearer, resulting in a higher transmittance (T) reading on the spectrophotometer. Transmittance represents the ratio of light that falls on a substance to the light that passes through it. A higher transmittance value indicates a larger amount of light passing through the substance. To conduct the assay, the collected blood samples were centrifuged for ten minutes at 5000 RPM to collect the plasma. The supernatant was collected from each sample and put into Eppendorf tubes and set aside. Then a suspension of *Micrococcus lysodeikticus* was made at a concentration of 0.2 mg/ mL in 0.05 M (pH = 6.2) sodium phosphate buffer. Then 1 mL of the suspension was added to an Eppendorf tube. 50 μL of the plasma was then added to the Eppendorf tube and vortexed. 1 mL of this solution was put into a cuvette to measure its transmittance at 540 nm using a spectrophotometer (Spectronic 601 spectrophotometer, Milton Roy Company, PA). Readings were taken at 1 minute and 5-minute mark. This procedure was repeated for each and every one of the sera collected from the fish. To calibrate the spectrophotometer, an uninoculated sodium phosphate buffer was used. We used the following formula to calculate the lysozyme activity assay,

LAA=(Finaltransmittance-Initialtransmittance)/Totalelapsedtime

Thus, the lysozyme activity assay is an indicator of rate of increase in transmittance per minute due to the lysozyme clearing bacteria.

### Statistical analysis

The collected data were analyzed using SigmaPlot^®^ 14.0, Systat Software Inc. A one-way analysis of variance (ANOVA) followed by Tukey’s test was performed to determine whether the differences between the treatment groups were significant (P < 0.05). The analyzed data is presented in the form of means ± standard errors of means (SEM) throughout the paper.

## Results and discussions

Due to lack of research on the effects of CBD on the stress physiology of fish, we have reviewed literature that has tested the effects of CBD on mice models whenever fish model research was unavailable. At the moment, the effects of CBD on the stress physiology of fish is a novel area of research. This makes our research one of the first to investigate the potential use of CBD as a stress-mitigating agent in fish, namely, O. *niloticus*. Whereas CBD on mice models have been well explored in this area. When discussing the effects of stress on animal physiology, we review the stress physiology of fish as it is a well-reviewed and known area of research.

Firstly, cortisol ELISA assay was done to see whether the hydrocortisone fed to the fish was able to stress the fish effectively. In order to interpret the results, we must first understand how cortisol is generated in the body and how long it remains active. In fish, stressors stimulate the hypothalamic-pituitary-interrenal axis (HPI) which prompts the release of adrenocorticotrophic hormones (ACTH). This in turn prompts the interrenal cells of the head kidney tissue to generate cortisol in response. This is similar to the cortisol production in mammals who have hypothalamic-pituitary-adrenal cortex (HPA) and are also stimulated by the adrenocorticotrophic hormones (ACTH) [29]. The cortisol release is controlled by a negative feedback loop at the HPI axis [30]. The plasma cortisol levels are kept in check by regulating how much of it is produced endogenously. In fish, the plasma cortisol levels come down to the basal levels 24 hours after an acute stressor is perceived. Cortisol is rapidly metabolized in the liver due to its action on it, and then filtered and excreted by the kidney [31].

**Fig 1:**
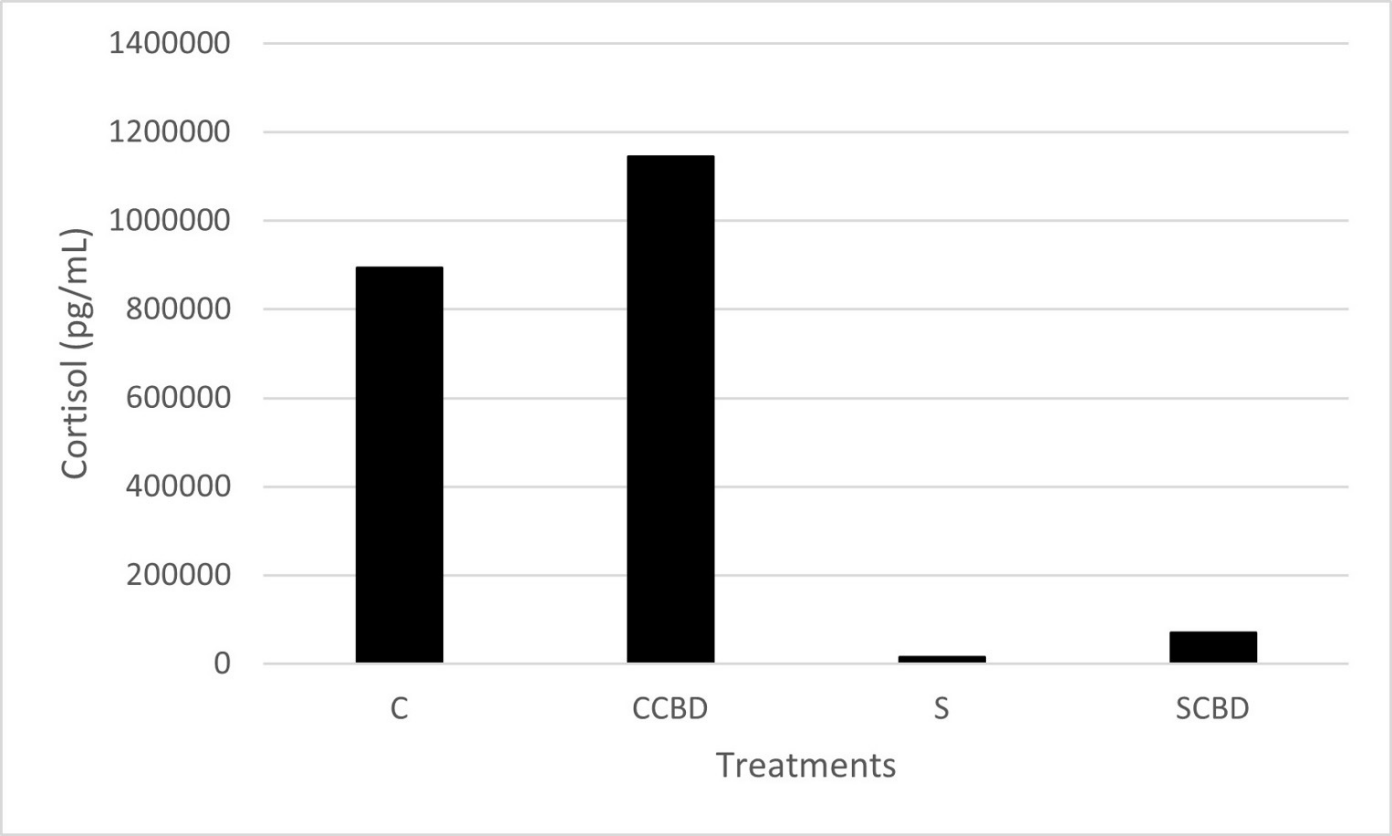
Plasma cortisol concentrations in pg/ mL of Nile tilapia fed with four different treatments represented by C (Control feed), CCBD (Control feed with CBD), S (Stress feed = feed with hydrocortisone) and SCBD (Stress feed with CBD = feed with hydrocortisone and CBD) groups. Results are presented as means and no standard error bar is presented as blood serum were pooled together from all the sample fish per treatment (N = 1).

According to Fig 1, the stress groups (S and SCBD) had a lower plasma cortisol level in comparison to the control groups (C and CCBD). Statistical analysis is not possible as the serum were pooled together from all the sample fish within the treatment group before running ELISA assay (N = 1). It may seem counterintuitive; however, our result is an indication that the exogenously supplemented cortisol via fish feed were getting into the blood of the fish and thus were stressing the fish. The increased level of exogenous plasma cortisol in turn was shutting down the endogenous cortisol production due to the negative feedback loop at the HPI axis [30]. Inhibition of the HPI axis would shut down the production of ACTH and thus inhibit the production of cortisol from the interrenal cells of the head kidney tissue. Then, the exogenous cortisol in the plasma were being metabolized rapidly by the liver and excreted completely within 24 hours. Blood cortisol is cleared at a faster rate when fish are administered with exogeneous cortisol in the blood to stimulate stress due to the increased level of cortisol in the blood [21]. The combined action of these systems lowered the plasma cortisol levels of the hydrocortisone supplement fed fish (S and SCBD) while keeping the endogenous cortisol levels of the control fed fish groups (C and CCBD) up. Since the control fish were not stressed using exogenous cortisol, they were able to maintain a basal level of endogenous cortisol throughout the 4 weeks of this experiment due to the HPI axis not shutting down via negative feedback loop and the regular rate of clearance of cortisol from the blood. Due to our dosage of hydrocortisone, each time after feeding, the fish would have had a spike in plasma cortisol which would manifest itself by affecting secondary or tertiary stress biomarkers down the line [21]. It is evident in our secondary and tertiary biomarkers. These findings are supported by previous studies [32] where similar phenomena were observed, such as in *O*. *niloticus* in high density stress conditions, cortisol fed catfish, *Ictalurus punctatus* [33] and *in-vitro* tissue culture of Coho salmon, *Oncorhynchus kisutch* [34].

**Fig 2:**
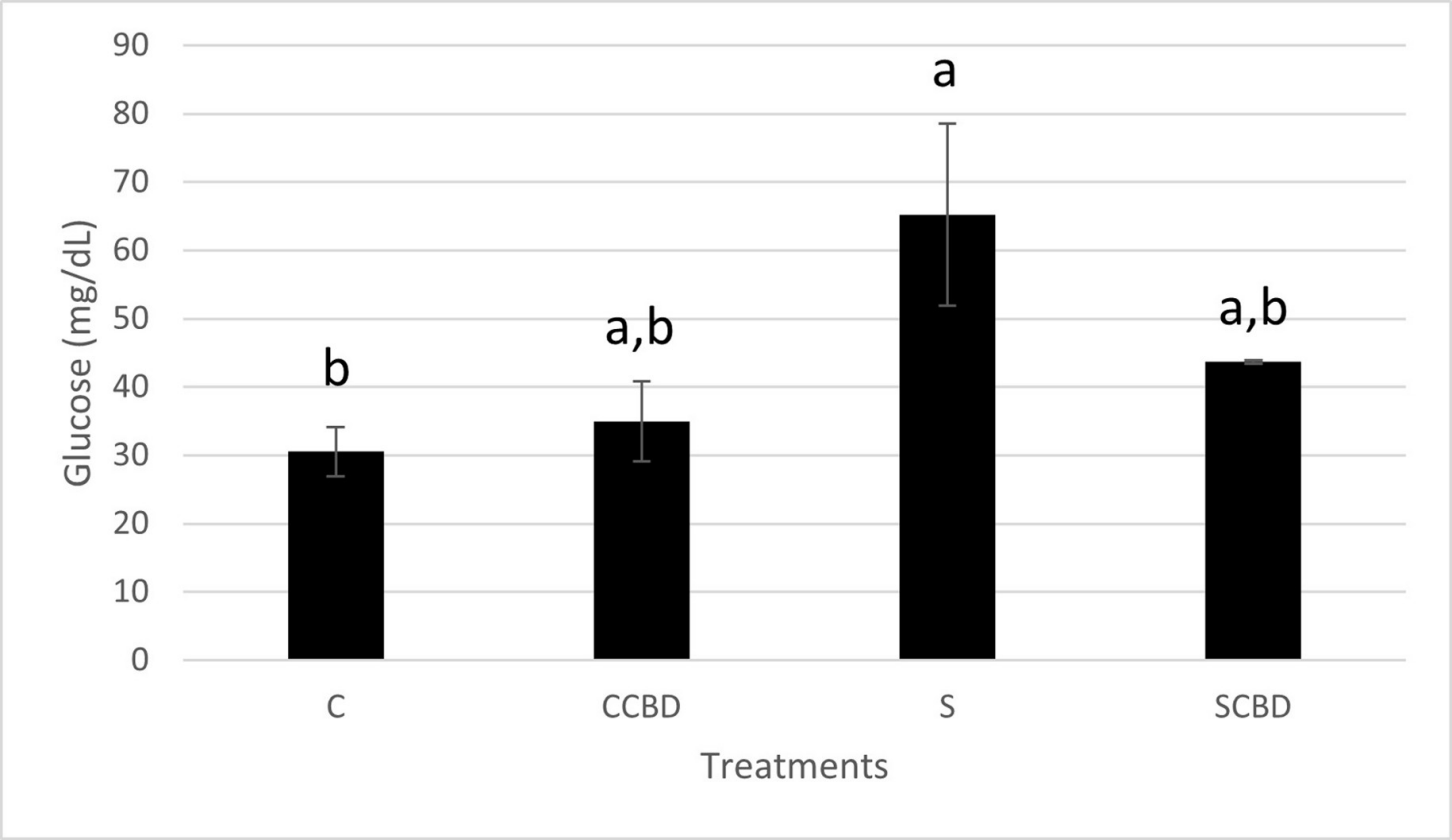
Blood glucose levels in mg/ dL of Nile tilapia fed with four different treatments represented by C (Control feed), CCBD (Control feed with CBD), S (Stress feed = feed with hydrocortisone) and SCBD (Stress feed with CBD = feed with hydrocortisone and CBD) groups. Results are presented as means± SEM. Different letters above the bar between groups denote Significant difference (P<0.05). N = 4, F = 3.709, DF = 3.

As we have now established that the stress fed treatment groups (S and SCBD) were in fact stressed, let us look at the physiological stress biomarkers and how they were affected by CBD. One of the most important secondary stress biomarkers is glucose. Stress increases the energy demands of the organism to fuel the fight or flight response and maintain homeostasis. This in turn demands for an increase in blood glucose to supply the energy demand. In order to do so, the pancreas releases glucagon which acts on the liver to break down glycogen into glucose [35]. Therefore, increased blood glucose is a sign of increased stress. In Fig 2 (N = 4, F = 3.709, DF = 3), the stressed group (S) had a significantly higher glucose level (more than double) than the control group (C; P = 0.046). Similar results have been reported on the blood glucose levels of fish under stress in various studies outlined by Martinez-Porchas et al. (2009). According to the paper, glucose and cortisol, along with a few other biomarkers of stress such as packed cell volume and protein can be good indicator of stress [36]. Also, in the case of stress groups (S vs SCBD), we see that the glucose level was lowered from 65 mg/ dL to 43 mg/ dL when CBD was mixed into the feed, although the results were not significantly different. Thus, CBD was somewhat effective in reducing blood glucose in stressed condition. The effect of CBD on mice blood glucose have been tested by Romero-Zerbo et al. in 2020. In the study, no significant difference was found in blood glucose between groups treated with CBD vs without CBD [37].

**Fig 3:**
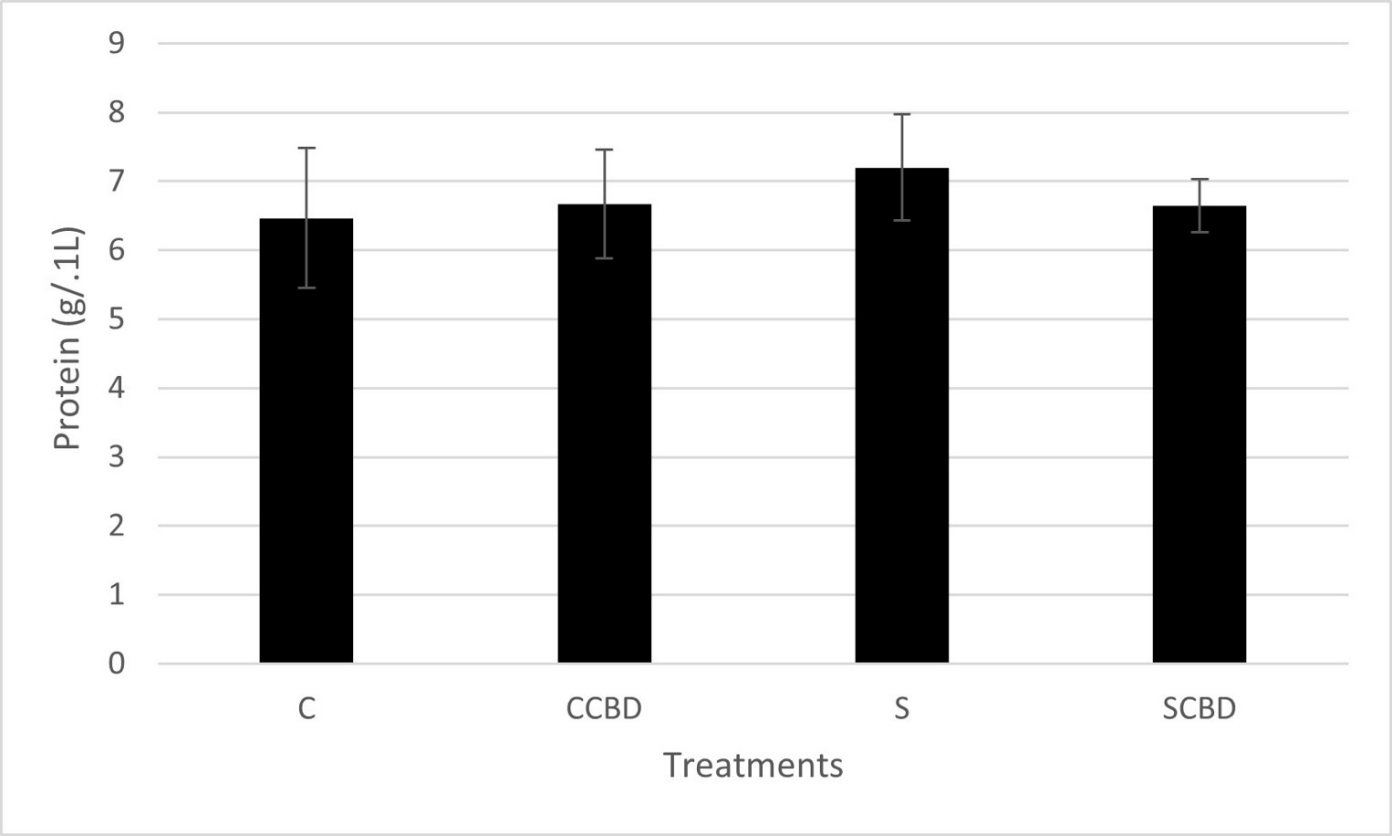
Plasma protein levels in g/ 0.1L of Nile tilapia fed with four different treatments represented by C (Control feed), CCBD (Control feed with CBD), S (Stress feed = feed with hydrocortisone) and SCBD (Stress feed with CBD = feed with hydrocortisone and CBD) groups. Results are presented as means± SEM. N = 4, P = 0.910, F = 0.176, DF = 3.

Another important secondary stress biomarker is the plasma protein level. As mentioned earlier, under stress, organisms increase their plasma protein levels to supply proteins around the body to repair any damage done to various tissues due to increased activity from stress [25]. So, under stressed conditions, higher protein levels are expected in the blood. In Fig 3 (N = 4, P = 0.910, F = 0.176, DF = 3), we see that all the protein levels across the treatments were at the same level (6.5–7.2 g/ 0.1 L). George Iwama (1998) in his paper titled “Stress in Fish” reviews among other things, the effect of stress on plasma proteins. In this paper, he reviews studies that show that stress does in fact increase blood plasma protein and this could be used as a stress biomarker. Plasma protein however, is not a reliable stress biomarker on its own and needs to be used alongside other biomarkers because of wide variability in results [38].

**Fig 4:**
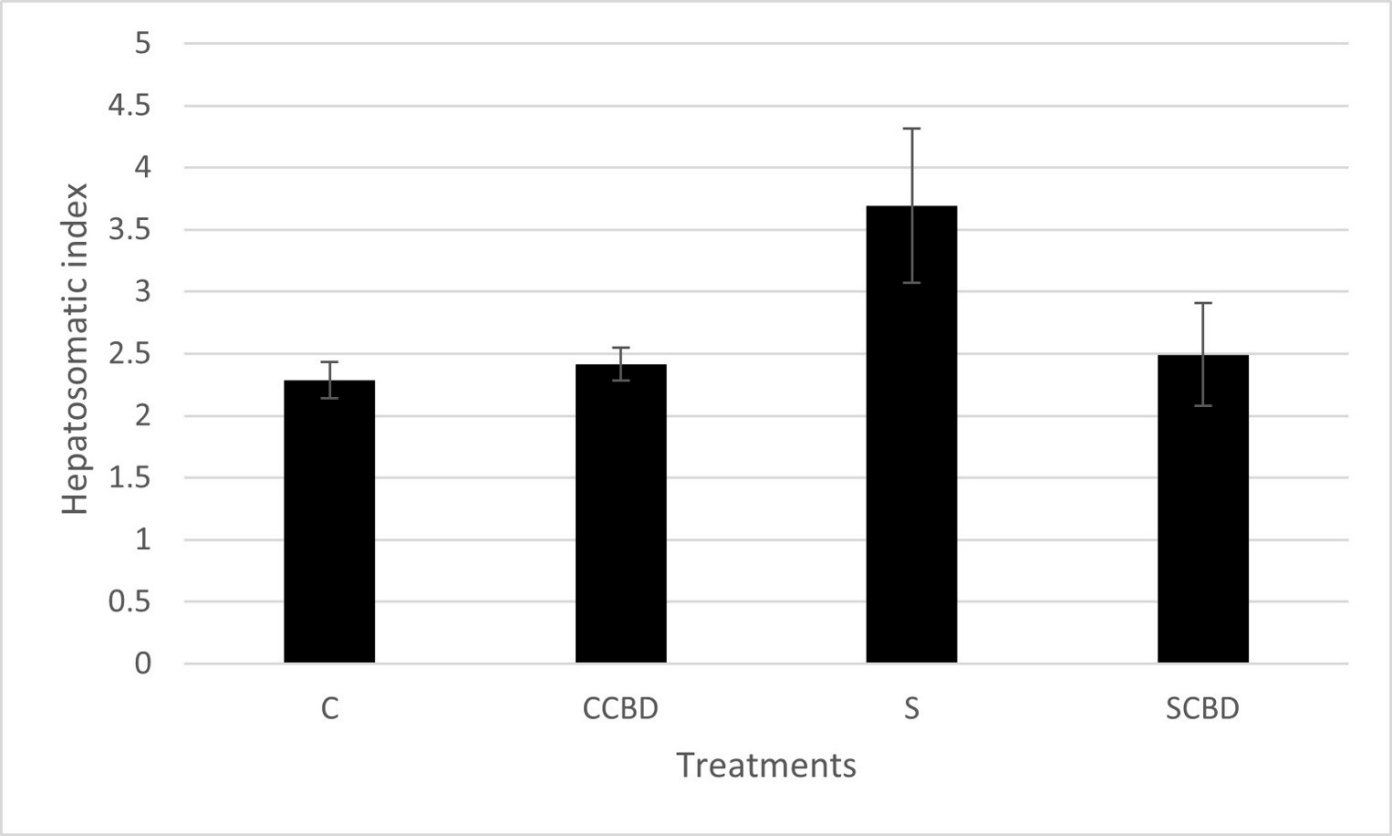
Hepatosomatic index of Nile tilapia fed with four different treatments represented by C (Control feed), CCBD (Control feed with CBD), S (Stress feed = feed with hydrocortisone) and SCBD (Stress feed with CBD = feed with hydrocortisone and CBD) groups. Results are presented as means± SEM. N = 4, P = 0.106, F = 2.589. DF = 3.

Continuing to look at the secondary stress biomarkers, hepatosomatic index (HSI) is the weight of the liver in proportion to the fish body weight. Hepatosomatic index should be low in organisms under acute stress as the glycogen stores in the liver get mobilized to form blood glucose under stress [21]. This glucose is then used to meet the high energy demands of a stressed condition as mentioned earlier. However, as stress is prolonged for a long time (chronic stress), the body stores energy as glycogen in the liver to deal with stress and this becomes the new basal HSI level. Higher metabolic activity in the liver due to stress can lead to higher HSI in chronic stress. This phenomenon is observed in previous research [39]. This phenomenon has also been observed in previous chronic stress studies (6 and 8 weeks) conducted in our lab on *O*. *niloticus* [40]. In Fig 4 (N = 4, P = 0.106, F = 2.589. DF = 3), it can be seen that stressed group (S) had the highest Hepatosomatic index at 3.65. This is much higher than all the other treatment groups (mean HSI of 2.25–2.5); however, this was not significantly different.

**Fig 5:**
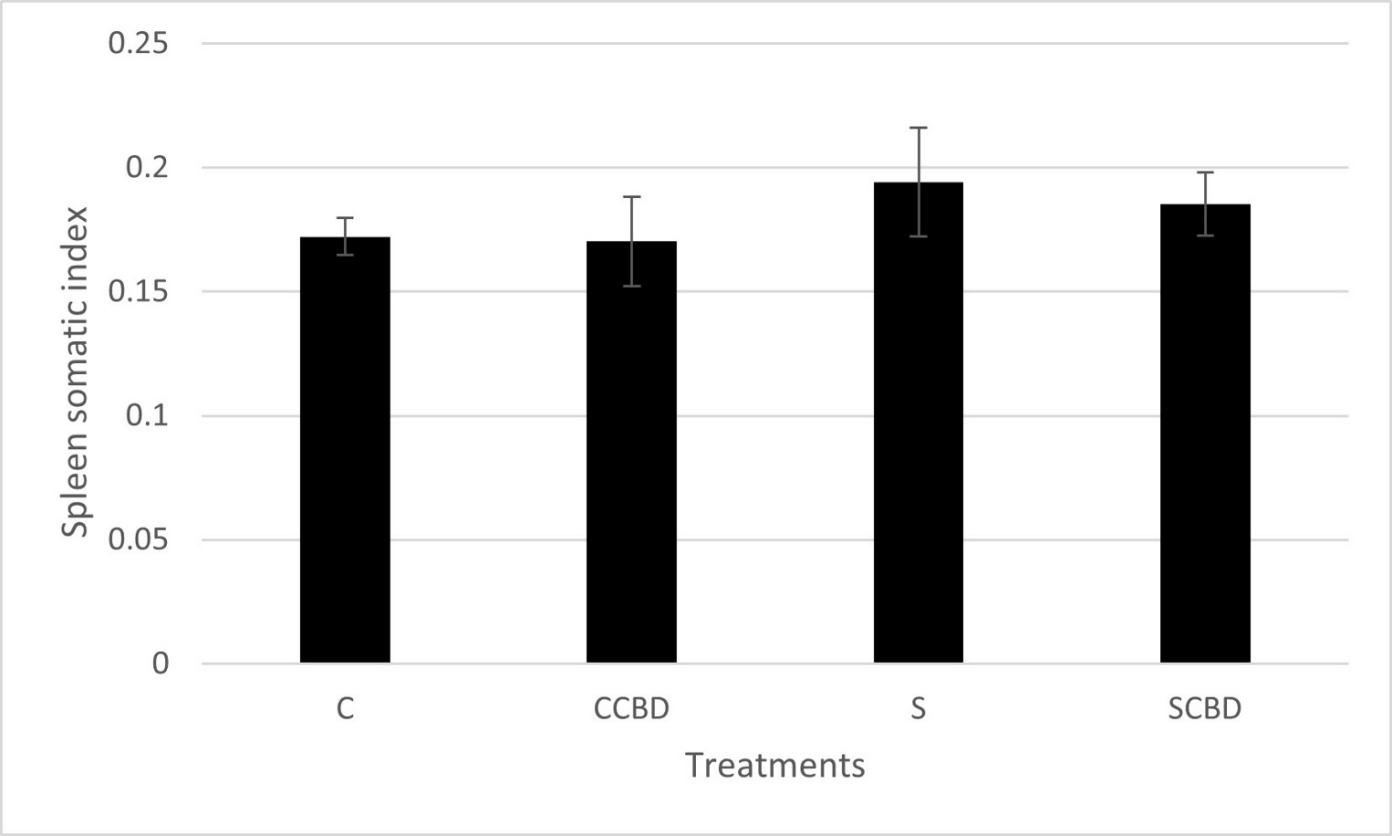
Spleen somatic index of Nile tilapia fed with four different treatments represented by C (Control feed), CCBD (Control feed with CBD), S (Stress feed = feed with hydrocortisone) and SCBD (Stress feed with CBD = feed with hydrocortisone and CBD) groups. Results are presented as means± SEM. N = 4, P = 0.695, F = 0.494, DF = 3.

Similar to the HSI, the spleen somatic index (SSI) measures the weight of the spleen in proportion to the fish’s total body weight, serving as an essential stress biomarker since the spleen stores immune cells and red blood cells [27]. Stress prompts the release of blood cells and spleen cells (T and B cells, macrophages) from the spleen to supply for the increased respiratory demands and to fight the perceived threat [27]. This results in a decrease in weight of the spleen in the short term [41]. However, in prolonged stress (chronic), just like in the case of the HSI, the spleen becomes larger to hold larger quantity of immune cells ready to be released in the case of another infection [42]. In a study published in 2017, it was found that mice exposed to chronic stress from crowding were more likely to physically bite each other. This in turn led to an increase in spleen weight over a period of 19 days due to the activation of spleen immune cells to fight off any invading pathogens from such bites [43, 44]. However, an increased production of immune cells may also affect the overall quality of the immune cells and their ability to fight infections [45]. Fig 5 (N = 4, P = 0.695, F = 0.494, DF = 3) shows that all the treatment groups had similar SSI levels between 0.175 and 0.19.

**Fig 6:**
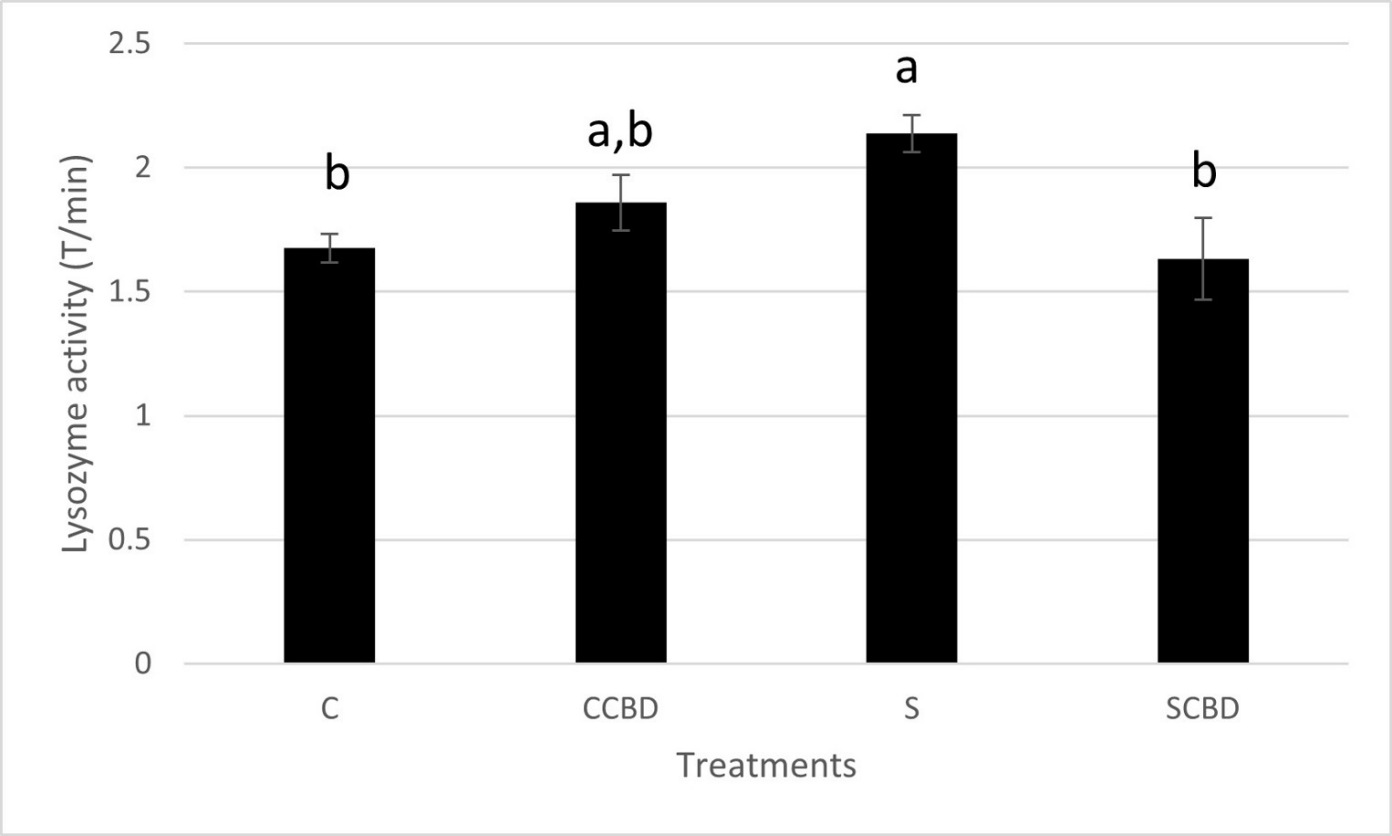
Lysozyme activity in Transmittance (T)/ minute of plasma of Nile tilapia fed with four different treatments represented by C (Control feed), CCBD (Control feed with CBD), S (Stress feed = feed with hydrocortisone) and SCBD (Stress feed with CBD = feed with hydrocortisone and CBD) groups. Results are presented as means± SEM. Different letters above the bar between groups denote Significant difference (P<0.05). N = 4, F = 4.613, DF = 3.

Finally, the tertiary stress biomarker, lysozyme activity demonstrates the ability of blood lysozyme to clear out bacterial solutions. Clearer the solution, higher the amount of light that passes through it in comparison to the light emitted by the spectrophotometer. Therefore, higher the transmittance (T). In stressed conditions, organisms produce increased amounts of lysozyme to fight off any perceived infectious threat [45]. This gives a higher transmittance value. Such is seen in Fig 6 (N = 4, F = 4.613, DF = 3), where the stressed group (S) had the highest transmittance value (2.1 T/ min), significantly higher (P = 0.046) than the control group (C) at 1.7 T/ min. This is indicative of stress. In another study it was demonstrated that handling stress in rainbow trout, *Oncorhynchus mykiss*, led to an increase in lysozyme activity of the fish [46]. Notably, between the stressed groups (S vs SCBD), CBD was able to reduce the lysozyme activity significantly (P = 0.029) from 2.1 to 1.6 T/ min (reduced by 1.3 times) to the control (C) level; this is indicative of a reduction in stress. This is not surprising as CBD is known to down regulate the immune system and act as an anti-inflammatory agent [47]. What is unknown is, whether the lowering of lysozyme activity seen in our data is due to CBD reducing stress by acting on the HPI axis or by directly affecting the immune system. Looking at all the other results alongside lysozyme activity, it is most likely a combination of both.

Lysozyme and SSI are both physiological and immunological parameters as they play a role in fish immunology. Lysozyme is responsible for clearing blood pathogens and spleen somatic index is tied in with the release of spleen cells responsible for immunomodulation. Comparing both Figs 5 and 6, we see an overlap in the trends. However, the differences among the treatment groups are much more profound in the lysozyme activity assay (Fig 6). Lysozyme secretion is more sensitive to stress and serves as a well-established biomarker of stress, indicating the ability of the immune system to respond to perceived threats. SSI is still important as it allows for the measurement of spleen weight compared to body weight and spleen health is important to the overall immune system, which is affected by stress.

## Conclusion

Overall, the stressed Nile tilapia group showed significantly higher values than the control group in two parameters, glucose, and lysozyme activity, indicating signs of stress .. Presence of CBD in the diet seems to mitigate stress in those two parameters. In the future, perhaps a higher dose of CBD supplementation for longer than four weeks may need to be tested to see its effects on further physiological parameters. It is important to know of the effects of CBD on the stress physiology and immunology of vertebrate organisms in a world where CBD is widely sold as a cure all, where it may or may not be the case. As this study demonstrates, CBD shows promise in reducing glucose and lysozyme activity caused by stress. Further investigation into the matter may not just be useful in stress mediation in aquatic organisms but may also have implications in human medicine as well.

## Supporting information

S1 File (XLSX)
